# Associations of *in Utero* Exposure to Perfluorinated Alkyl Acids with Human Semen Quality and Reproductive Hormones in Adult Men

**DOI:** 10.1289/ehp.1205118

**Published:** 2013-01-28

**Authors:** Anne Vested, Cecilia Høst Ramlau-Hansen, Sjurdur Frodi Olsen, Jens Peter Bonde, Susanne Lund Kristensen, Thorhallur Ingi Halldorsson, Georg Becher, Line Småstuen Haug, Emil Hagen Ernst, Gunnar Toft

**Affiliations:** 1 Danish Ramazzini Centre, Department of Occupational Medicine, Aarhus University Hospital, Aarhus, Denmark; 2 Department of Public Health, Section of Epidemiology, University of Aarhus, Aarhus, Denmark; 3 Centre for Fetal Programming, Statens Serum Institut, Copenhagen, Denmark; 4 Department of Occupational and Environmental Medicine, Bispebjerg Hospital of Copenhagen University, Copenhagen, Denmark; 5 Faculty of Food Science and Nutrition, University of Iceland, Reykjavik, Iceland; 6 Division of Environmental Medicine, Norwegian Institute of Public Health, Oslo, Norway; 7 Reproductive Laboratory, Institute of Anatomy, University of Aarhus, Aarhus, Denmark

**Keywords:** PFOA, PFOS, prenatal exposure, reproductive hormones, semen quality

## Abstract

Background: Perfluorinated alkyl acids (PFAAs), persistent chemicals with unique water-, dirt-, and oil-repellent properties, are suspected of having endocrine-disrupting activity. The PFAA compounds perfluorooctanoic acid (PFOA) and perfluorooctane sulfonic acid (PFOS) are found globally in humans; because they readily cross the placental barrier, *in utero* exposure may be a cause for concern.

Objectives: We investigated whether *in utero* exposure to PFOA and PFOS affects semen quality, testicular volume, and reproductive hormone levels.

Methods: We recruited 169 male offspring (19–21 years of age) from a pregnancy cohort established in Aarhus, Denmark, in 1988–1989, corresponding to 37.6% of the eligible sons. Each man provided a semen sample and a blood sample. Semen samples were analyzed for sperm concentration, total sperm count, motility, and morphology, and blood samples were used to measure reproductive hormones. As a proxy for *in utero* exposure, PFOA and PFOS were measured in maternal blood samples from pregnancy week 30.

Results: Multivariable linear regression analysis suggested that *in utero* exposure to PFOA was associated with lower adjusted sperm concentration (*p*_trend_ = 0.01) and total sperm count (*p*_trend_ = 0.001) and with higher adjusted levels of luteinizing hormone (*p*_trend_ = 0.03) and follicle-stimulating hormone (*p*_trend_ = 0.01). PFOS did not appear to be associated with any of the outcomes assessed, before or after adjustment.

Conclusions: The results suggest that *in utero* exposure to PFOA may affect adult human male semen quality and reproductive hormone levels.

Perfluorinated alkyl acids (PFAAs) are a class of chemicals with unique water-, dirt-, and oil-repellent properties; high stability; and resistance to degradation. They are used as surfactants in many industrial processes and consumer products, such as oil and water repellents for fabrics and food-packaging materials (Kissa 2001). Several PFAAs accumulate in food chains and have been detected in human serum worldwide (Giesy and Kannan 2001; Kannan et al. 2004). Although sources of human exposure are not fully understood, dietary intake is thought to be a major pathway of exposure in general populations, originating either from environmental contamination or migration from food packaging (Tittlemier et al. 2006, 2007). Furthermore, human exposure through drinking water in contaminated areas and dust in indoor environments may be significant (Emmett et al. 2006; Haug et al. 2011; Shoeib et al. 2011).

Two of the most abundant PFAAs in human serum samples are perfluorooctanoic acid (PFOA) and perfluorooctane sulfonic acid (PFOS). The half-lives of PFOA and PFOS in human serum have been estimated to be 3.8 years and 5.4 years, respectively (Olsen et al. 2007). Studies of adult male rats showed that PFOA exposure may cause reduced testosterone levels and increased estradiol levels (Lau et al. 2007), and a study on sexually mature mice indicated that PFOS exposure might affect testicular signalling, causing reduced serum testosterone and decreases in epididymal sperm counts (Wan et al. 2011). Two cross-sectional studies reported negative associations of PFOS, or high PFOA and PFOS combined, with the proportion of morphologically normal spermatozoa in adult men (Joensen et al. 2009; Toft et al. 2012). Furthermore, in a study of men attending an *in vitro* fertilization clinic, Raymer et al. (2012) reported that luteinizing hormone (LH) and free testosterone were significantly positively correlated with plasma PFOA, although PFOA was not associated with semen characteristics.

Because of the widespread environmental occurrence of PFOA and PFOS, along with their ability to cross the placental barrier (Fei et al. 2007; Gützkow et al. 2012; Inoue et al. 2004), exposure of the developing human fetus to these compounds is inevitable. This is of concern because fetal development of the male reproductive organs may be disturbed by exposure to exogenous factors (Jensen et al. 2010; Palmer et al. 2009). In addition, rat studies have suggested the existence of a male programming window—corresponding to gestational weeks 8–14 in humans—during which xenobiotic exposure may affect reproductive hormone balance and impact normal male reproductive development (Welsh et al. 2008). To our knowledge, potential associations between prenatal exposure to PFOA and PFOS and adverse effects on the human male reproductive system have not been investigated, although potential effects on immune system development were studied by Grandjean et al. (2012), who reported decreased antibody responses to childhood diphtheria vaccinations in association with prenatal PFOA and PFOS exposures.

The prospective design of the present study enables us to investigate the hypothesis that *in utero* exposure to PFOA and PFOS is associated with reduced semen quality and testicular size, as well as altered reproductive hormones, in adult men.

## Methods

*Population*. Physical examinations were performed on sons of a pregnancy cohort recruited to a study in 1988–1989 in Aarhus, Denmark. Mothers answered a questionnaire on dietary and lifestyle habits and provided a blood sample, which was stored in a biobank at –20°C. Of all invited pregnant women, 80% participated (*n* = 965) (Olsen et al. 1995a, 1995b). In 2008, sons of the pregnancy cohort were invited to answer an Internet-based questionnaire on health and lifestyle habits, and in 2008–2009 they were asked to undergo a physical examination in which they donated a semen sample (which they had produced at home), gave a blood sample to be analyzed for reproductive hormones, and self-measured testicular volumes (Vested et al. 2011). Of the 468 sons invited to complete the questionnaire, 176 consented to the physical examination, corresponding to a participation rate of 37.6% of the eligible cohort of sons. Each man provided written informed consent prior to participation, and the study was approved by the Central Denmark Region Committees on Health Research Ethics (registration number M-20070157).

**Figure 1:**
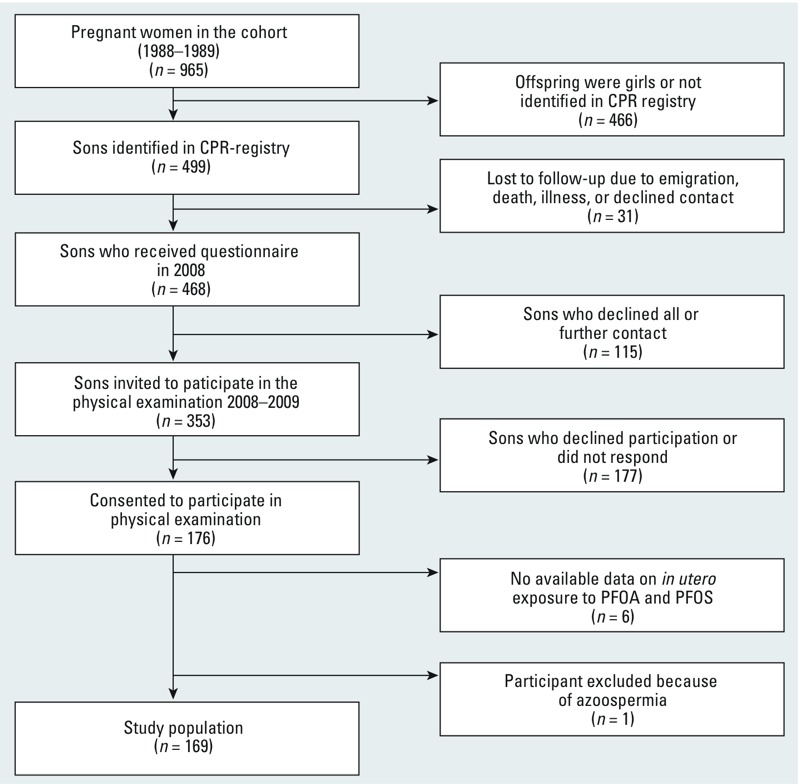
Flow chart of recruitment to the physical examination of the follow-up study.

Maternal measurements for PFOA and PFOS serum concentrations were missing for six participants, and one was excluded from all statistical analyses due to azoospermia. Hence, the study population included 169 men (Figure 1).

*Semen collection and physical examination*. Physical examinations were conducted from February 2008 to September 2009. Semen samples were stored in a heating chamber (37°C) until analysis. All participants provided information on time and date of the semen sample collection and spillage during semen sample collection. Testicular volume self-measurements were performed using a Prader orchidometer, a method reported to be valid for testicular size measurement (Ramlau-Hansen et al. 2007). The examiner measured the height and weight of each participant. Blood samples were taken between 0730 hours and 1330 hours.

*Semen analysis*. For 86% of the samples, semen analysis was initiated within 1 hr of ejaculation; all semen samples were analyzed within 2 hr. We estimated semen volume based on weight (1 g = 1 mL). Conventional analysis of motility and sperm concentration was performed by two laboratory technicians (one in the beginning and the other at the end of the study) according to World Health Organization guidelines (World Health Organization 1999). Motility, sperm concentration, and total sperm counts were also assessed by computer-assisted semen analysis (CASA) using CRISMAS Clinical software, version 4.6 (Image House Medical, IHMedical A/S, Copenhagen, Denmark), as previously described (Vested et al. 2011). The laboratory continuously participated in the European Society for Human Reproduction and Embryology external quality control program. All tests were in agreement with the quality control standards. Sperm morphology was classified as normal or abnormal using “strict” criteria as described by Menkveld et al. (1990).

*Blood sample analysis*. Maternal serum samples were collected in pregnancy week 30 in 1988–1989 and stored at –20°C until analysis in 2010–2011. Concentrations of PFOA and PFOS were determined by column-switching, isotope dilution liquid chromatography–tandem mass spectrometry at the Division of Environmental Medicine, Norwegian Institute of Public Health (Oslo, Norway), as previously described (Haug et al. 2009). Limits of quantification were 0.05 ng/mL. Quality of the analytical procedure was monitored by analyzing in-house quality control samples (*n* = 18) and human serum samples from an interlaboratory comparison exercise (*n* = 3). Coefficients of variation (CVs) for PFOA and PFOS for the in-house quality control samples were 11% and 4.4%, respectively, and results of the interlaboratory comparison were within 1 SD of the consensus values.

Serum concentrations of sex hormone-binding globulin (SHBG) were measured using a solid-phase two-site chemiluminescent immunometric assay (IMMULITE® 2000; Siemens Healthcare Diagnostics Products Ltd., Gwynedd, UK) with CVs of 4.5–4.7%; concentrations of LH, follicle-stimulating hormone (FSH), estradiol, and testosterone were analyzed by immunoassays (cobas® 6000 e601; Roche Diagnostics, Mannheim, Germany) at the Department of Clinical Biochemistry, Aarhus University Hospital, with CVs of 1.1–2.4%, 1.9–2.1%, 1.5–2.9%, and 2.2–4.5%, respectively. Undiluted inhibin-B was measured at the Laboratory of Reproductive Biology, Juliane Marie Centre for Women, Children and Reproduction (University Hospital of Copenhagen, Copenhagen, Denmark) using a commercially available ELISA (Oxford Bio-innovation Ltd., Oxfordshire, England, UK) with a detection limit of 20 pg/mL and a CV < 7%. Measurements below the detection limit for LH (*n* = 1), FSH (*n* = 1), and estradiol (*n* = 4) were recoded to half the detection limit (0.15 IU/L, 0.15 IU/L, and 0.025 nmol/L, respectively).

*Statistical analysis*. Outcome variables included semen parameters [sperm concentration, total sperm count, semen volume, percentage progressive spermatozoa (rapidly progressive + slowly progressive), and percentage morphologically normal spermatozoa], mean testicular volume (estimates were comparable for left and right volumes separately), and reproductive hormones (testosterone, estradiol, LH, FSH, SHBG, and inhibin B). Free androgen index (FAI) was calculated as (testosterone/SHBG) × 100.

Differences across tertiles of maternal PFOA and PFOS exposure were estimated using one-way analysis of variance (ANOVA) tests and chi-square tests. We tested crude trends by Spearman’s rank correlation test and adjusted trends by multivariable regression analyses by entering PFOA and PFOS as continuous variables in the model. Outcome variables were natural logarithm (ln)-transformed before multivariable regression analysis, and participants were divided into three groups according to tertiles of maternal PFOA [low (1.26–3.15 ng/mL), medium (≥ 3.15–4.40 ng/mL), and high (≥ 4.40–16.57 ng/mL)] and PFOS [low (7.47–18.78 ng/mL), medium (≥ 18.78–24.31 ng/mL), and high (≥ 24.31–54.28 ng/mL)] concentrations, or according to quintiles (for associations with a significant linear trend). Differences between the two upper tertiles versus the lowest tertile were tested by two-sample Wilcoxon rank-sum (Mann–Whitney) test (crude results) and by multivariable regression analysis for each of the outcome variables, with low PFOA and PFOS groups as referents. Results are presented as adjusted percentage differences with 95% confidence intervals (CIs), which were calculated based on the log scale output from the regression analysis. Multivariable regression results were adjusted for the following *a priori* selected potential confounders: history of reproductive tract disease [cryptorchidism, hypospadias, inguinal hernia, varicocele, testicular hydrocele, incarcerated hernia, phimosis, testicular torsion, chlamydia, gonorrhea, and epididymitis, combined into one dichotomous variable (any versus none)], body mass index (continuously in kilograms per meter squared), smoking status (current and party smoker/exsmoker and never smoker), smoking by the participant’s mother during pregnancy (yes/no), and socioeconomic status at birth [total annual income for the household in 1987 < 200,000 DKK (kroner) or ≥ 200,000 DKK]. In addition, sperm concentration, total sperm count, percentage progressive spermatozoa, semen volume, and testicular volume were adjusted for abstinence time (≤ 48 hr, 49–120 hr, ≥ 121 hr); sperm concentration for spillage (yes/no); percentage progressive spermatozoa for time from ejaculation to semen analysis (continuous, in minutes); and reproductive hormones for time of day of blood sampling (0730–0929 hours, 0930–1129 hours, later than 1130 hours). Participants reporting spillage during semen sample collection (*n* = 45) were excluded from the analyses of total sperm count and semen volume.

All statistical analyses were performed using Stata 11.2 software (StataCorp, College Station, TX, USA), and a two-tailed probability level of *p* < 0.05 was considered statistically significant.

## Results

The 169 study participants had a median age of 20 years (range, 19–21 years). The mothers’ median (25th–75th percentile) plasma concentrations of PFOA and PFOS were 3.8 ng/mL (2.8–4.7 ng/mL) and 21.2 ng/mL (17.4–26.5 ng/mL), respectively, and PFOA and PFOS concentrations were highly correlated (Spearman’s rho = 0.73; *p* < 0.0001).

Characteristics of the study participants according to tertiles of *in utero* PFOA and PFOS exposure are shown in Table 1. Characteristics did not differ substantially.

**Table 1:** Characteristics of participants (*n* = 169) and biologic samples according to tertiles of PFOA and PFOS concentrations in maternal serum at gestational week 30.

Characteristic	PFOA				PFOS			
	Low (n = 57)	Medium (n = 56)	High (n = 56)	p-Value	Low (n = 57)	Medium (n = 56)	High (n = 56)	p-Value
Person-related characteristics of sons								
Body mass index (kg/m2; mean ± SD)	22.7 ± 2.6	23.1 ± 3.1	22.5 ± 3.0	0.6a	22.8 ± 2.4	22.6 ± 2.9	23.0 ± 3.4	0.7a
History of reproductive tract diseaseb	6 (10.5)	11 (19.6)	8 (14.3)	0.7c	5 (8.8)	9 (16.1)	11 (19.6)	0.7c
Current/occasional smoker	26 (45.6)	28 (50.0)	28 (50.0)	0.5c	29 (50.9)	30 (53.6)	23 (41.1)	0.5c
Person-related characteristics of mothers								
Mother smoked during pregnancy	20 (35.1)	22 (39.3)	11 (19.6)	0.1c	22 (38.6)	18 (32.1)	13 (23.2)	0.3c
Socioeconomic status (total annual household income 1987; DKK)								
< 200,000	23 (40.4)	21 (37.5)	16 (28.6)	0.6c	20 (35.1)	23 (41.1)	17 (30.4)	0.8c
≥ 200,000	31 (54.4)	29 (51.8)	35 (62.5)	0.6c	32 (56.1)	28 (50.0)	35 (62.5)	0.8c
Semen and blood-related characteristics								
Duration of abstinence								
≤ 48 hr	34 (59.7)	27 (48.2)	31 (55.4)	0.6c	37 (64.9)	25 (44.6)	30 (53.6)	0.1c
49 hr–5 days	22 (38.6)	25 (44.6)	23 (41.1)	0.6c	20 (35.1)	27 (48.2)	23 (41.1)	0.1c
> 5 days	1 (1.8)	4 (7.1)	2 (3.6)	0.6c	0 (0)	4 (7.1)	3 (5.4)	0.1c
Minutes from ejaculation to semen analysis (mean ± SD)	45.9 ± 16.1	41.9 ± 21.2	41.2 ± 20.5	0.4a	42.0 ± 17.0	45.1 ± 19.9	42.2 ± 21.1	0.7a
Spillage occurred at semen sampling	16 (28.1)	18 (32.1)	11 (19.6)	0.4c	16 (28.1)	14 (25.0)	15 (26.8)	0.9c
Time blood was sampled								
0730–0929 hours	16 (28.1)	22 (39.3)	16 (28.6)	0.1c	18 (31.6)	16 (28.6)	20 (35.7)	0.2c
0930–1129 hours	28 (49.1)	23 (41.1)	32 (57.1)	0.1c	22 (38.6)	32 (57.1)	29 (51.8)	0.2c
Later than 1130 hours	13 (22.8)	8 (14.3)	8 (14.3)	0.1c	15 (26.3)	7 (12.5)	7 (12.5)	0.2c
Values are n (%) unless otherwise stated. Tertiles are as follows: for PFOA, low (1.26–3.15 ng/mL), medium (≥ 3.15–4.40 ng/mL), and high (≥ 4.40–16.57 ng/mL); for PFOS, low (7.47–18.78 ng/mL), medium (≥ 18.78–24.31 ng/mL), and high (≥ 24.31–54.28 ng/mL). aOne-way ANOVA test of differences across tertiles of maternal PFOA and PFOS exposure. bIncludes cryptorchidism, hypospadias, inguinal hernia, varicocele, testicular hydrocele, incarcerated hernia, phimosis, testicular torsion, chlamydia, gonorrhea, and epididymitis, combined into one variable (yes/no). cChi-square test of differences across tertiles of maternal PFOA and PFOS exposure.								

Trend tests on crude sperm concentration and total count did not indicate significant associations with PFOA exposure (Table 2). However, estimates from multivariable regression models indicated significant negative trends for sperm concentration and total sperm count associated with *in utero* exposure to PFOA, as well as a 34% reduction (95% CI: –58, 5%) in sperm concentration and a 34% reduction (95% CI: –62, 12%) in total sperm count estimated for the highest exposure group compared with the lowest (Table 2). A subanalysis of associations with quintiles of PFOA exposure suggested that the statistically significant negative linear trends for sperm concentration and total sperm count were largely driven by pronounced decreases of sperm concentration and total sperm count among men in the 5th quintiles [see Supplemental Material, Table S1 (http://dx.doi.org/10.1289/ehp.1205118)]. CASA using CRISMAS Clinical software supported the results from the manual assessment of semen analysis (see Supplemental Material, Table S2). CASA results indicated that men in the high-PFOA tertile had 33% (95% CI: –54, –1%) lower sperm concentrations and 34% (95% CI: –58, 6%) lower total sperm counts compared with men in the low-PFOA group.

**Table 2:** Semen, testicular size, and reproductive hormone characteristics for 169 young Danish men stratified by tertiles of maternal serum PFOA concentrations at pregnancy week 30.

Parameter	n	Median (25th–75th percentile)			Spearman’s rhoa	ptrenda	Percent difference from low PFOA (95% CI)		Adjusted β (SE)c	Adjusted ptrendc
		Low PFOA	Medium PFOA	High PFOA			Medium PFOAb	High PFOAb		
Sperm concentration (million/mL)	168	33 (23–59)	46 (14–71)	30 (10–66)	–0.11	0.15	–7 (–42, 47)	–34 (–58, 5)	–0.11 (0.04)	0.01
Total sperm count (million)	123	121 (59–187)	144 (59–204)	74 (31–223)	–0.15	0.10	2 (–42, 81)	–34 (–62, 12)	–0.20 (0.06)	0.001
Semen volume (mL)	123	3 (2– 4)	3 (2–4)	3 (2–4)	0.09	0.34	14 (–8, 41)	12 (–8, 37)	–0.01 (0.02)	0.54
Percentage progressive spermatozoa	167	67 (60–77)	60 (51–70)	66 (52–72)	–0.14	0.08	–9 (–17, 1)	–8 (–16, 2)	–0.02 (0.01)	0.10
Percentage morphologically normal spermatozoa	152	9 (5–13)	7 (4–12)	9 (4–13)	–0.05	0.54	–24 (–45, 6)	–19 (–42, 13)	–0.05 (0.03)	0.13
Mean testicular volume (mL)	168	15 (12–20)	15 (11–20)	15 (11–19)	–0.06	0.41	1 (–12, 16)	–6 (–18, 8)	–0.01 (0.01)	0.62
Testosterone (nmol/L)	169	22 (18–25)	21 (17–24)	21 (18–26)	–0.03	0.70	–2 (–13, 10)	1 (–10, 12)	0.00 (0.01)	0.70
Estradiol (nmol/L)	169	0.09 (0.08–0.11)	0.09 (0.08–0.12)	0.10 (0.08–0.12)	0.11	0.15	1 (–11, 15)	7 (–6, 21)	0.02 (0.01)	0.17
LH (IU/L)	169	4.2 (3.1–5.7)	4.2 (3.1–5.2)	4.7 (3.8–5.7)	0.12	0.11	6 (–11, 27)	24 (4, 48)	0.04 (0.02)	0.03
FSH (IU/L)	169	2.6 (1.8–3.8)	3.1 (2.4–4.2))	3.3 (2.4–4.8)	0.17	0.03	15 (–8, 44)	31 (5, 64)	0.06 (0.02)	0.01
Inhibin B (pg/mL)	169	224 (172–258)	213 (153–278)	223 (169–262)	–0.02	0.82	2 (–14, 21)	0 (–15, 18)	–0.02 (0.02)	0.19
SHBG (nmol/L)	169	26 (22–34)	26 (20–33)	26 (22–33)	–0.03	0.72	–4 (–16, 10)	–3 (–15, 12)	–0.01 (0.01)	0.44
FAI	169	78 (67–94)	82 (66–101)	81 (67–99)	0.02	0.85	2 (–11, 17)	3 (–10, 18)	0.01 (0.01)	0.66
The number of participants in each regression analysis depended on the outcome variable and missing data in the covariates. aSpearman’s rho and p-value for PFOA (continuous) and untransformed outcomes. bAll multivariable regression results were adjusted for history of reproductive tract disease, son’s body mass index, son’s smoking status, maternal smoking during pregnancy, and socioeconomic status. Sperm concentration, total sperm count, progressive spermatozoa, semen volume, and testicular volume were adjusted for abstinence time; sperm concentration was also adjusted for spillage during semen sample collection; progressive spermatozoa was also adjusted for time from ejaculation to semen analysis; reproductive hormones were also adjusted for time of day of blood sampling. cAdjusted β-coefficient for PFOA modeled as a continuous variable in a multivariable linear regression model of ln-transformed outcomes, with adjustment for covariates as indicated above and p-value as a test of linear trend.										

No significant trends of associations between the percentage of progressive spermatozoa and PFOA were indicated in either crude or adjusted results based on the manual assessment (Table 2). However, in the comparison between tertiles of exposure, crude results showed that sons in the medium-PFOA group had 10% fewer progressive spermatozoa than those in the low-PFOA group (*p* = 0.02). After adjustment, this association was no longer statistically significant (*p* = 0.06). CASA semen analysis provided somewhat stronger indications of an association between PFOA exposure and the percentage of progressive spermatozoa, with a significant trend in the adjusted analyses and a 13% relative decrease (95% CI: –23, –2%) in the percentage of progressive motile spermatozoa in the high-PFOA group compared with the low-PFOA group [see Supplemental Material, Table S2 (http://dx.doi.org/10.1289/ehp.1205118)].

For associations of *in utero* exposure to PFOA and sperm morphology, semen volume, or testicular volume, we observed no significant trends or differences between PFOA exposure groups (Table 2).

For the reproductive hormones, we observed a trend of higher crude FSH levels with higher PFOA exposure, which remained statistically significant after transformation and adjustment (Table 2). Based on the crude analysis of differences between tertiles, sons in the high-PFOA group had 27% higher FSH levels (*p* = 0.03) than sons in the low-PFOA group (Table 2). After adjustment, estimated FSH levels were 31% higher (95% CI: 5, 64%) in the high-PFOA group than in the low-PFOA group. LH was not statistically significantly associated with PFOA exposure based on the crude trend test; however, adjusted analyses showed a trend of higher LH with higher *in utero* exposure to PFOA, with a 24% (95% CI: 4, 48%) higher LH level estimated for the high- versus low-PFOA exposure group. We observed no statistically significant associations between *in utero* exposure to PFOA and any of the other reproductive hormones or FAI (Table 2).

Analysis of *in utero* exposure to PFOS showed no significant trends or associations for any of the measured semen outcomes or reproductive hormones in either crude or adjusted analyses [Table 3; see also Supplemental Material, Table S3 (http://dx.doi.org/10.1289/ehp.1205118)]. A subanalysis adjusting PFOA trend analyses for PFOS did not alter the observed associations significantly (data not shown).

**Table 3:** Semen, testicular size, and reproductive hormone characteristics for 169 young Danish men stratified by tertiles of maternal serum PFOS concentrations at pregnancy week 30.

Parameter	n	Median (25th–75th percentile)			Spearman’s rhoa	ptrenda	Percent difference from low PFOS (95% CI)		Adjusted β (SE)c	Adjusted ptrendc
		Low PFOS	Medium PFOS:	High PFOS			Medium PFOSb	High PFOSb		
Sperm concentration (million/mL)	168	35 (19–58)	32 (12–62)	37 (17–94)	0.00	0.99	–24 (–52, 21)	–1 (–38, 59)	–0.01 (0.01)	0.37
Total sperm count (million)	123	103 (55–176)	77 (42–204)	124 (50–244)	0.01	0.87	–36 (–64, 12)	–23 (–56, 38)	–0.02 (0.01)	0.12
Semen volume (mL)	123	3 (2–4)	3 (2–4)	3 (2–4)	0.05	0.56	–8 (–26, 13)	–5 (–24, 18)	0.00 (0.01)	0.58
Percentage progressive spermatozoa	167	66 (57–74)	67 (54–76)	63 (52–70)	–0.13	0.10	0 (–9, 10)	–7 (–16, 2)	0.00 (0.00)	0.17
Percentage morphologically normal spermatozoa	152	9 (4–13)	8 (4–14)	9 (4–12)	–0.05	0.57	–4 (–31, 34)	–14 (–39, 20)	–0.01 (0.01)	0.31
Mean testicular volume (mL)	168	15 (12–20)	14 (11–20)	15 (12–20)	–0.03	0.69	–9 (21, 4)	–4 (–17, 11)	0.00 (0.00)	0.52
Testosterone (nmol/L)	169	22 (19–26)	20 (16–24)	21 (18–25)	–0.05	0.50	–10 (–19, 1)	–5 (–15, 6)	0.00 (0.00)	0.87
Estradiol (nmol/L)	169	0.10 (0.08–0.12)	0.09 (0.08–0.11)	0.1 (0.08–0.12)	0.06	0.45	–7 (–19, 5)	1 (–11, 16)	0.00 (0.00)	0.27
LH (IU/L)	169	4 (4–6)	4 (3–5)	5 (4–6)	0.00	0.93	–7 (–22, 12)	–2 (–18, 18)	0.00 (0.00)	0.95
FSH (IU/L)	169	3 (2– 4)	3 (2–4)	3 (2–5)	0.12	0.13	3 (–18, 29)	20 (–5, 51)	0.01 (0.01)	0.06
Inhibin B (pg/mL)	169	225 (163–256)	221 (148–274)	214 (171–266)	–0.01	0.91	–6 (–20, 11)	0 (–16, 19)	0.00 (0.00)	0.72
SHBG (nmol/L)	169	26 (22–35)	25 (20–30)	29 (22–36)	0.01	0.89	–10 (–22, 2)	5 (–8, 20)	0.00 (0.00)	0.66
FAI	169	81 (67–103)	84 (68–98)	77 (66–94)	–0.03	0.68	1 (–12, 16)	–10 (–21, 4)	0.00 (0.00)	0.57
The number of participants in each regression analysis depended on the outcome variable and missing data in the covariates. aSpearman’s rho and p-value for PFOS (continuous) and untransformed outcomes. bAll multivariable regression results were adjusted for history of reproductive tract disease, son’s body mass index, son’s smoking status, maternal smoking during pregnancy, and socioeconomic status. Sperm concentration, total sperm count, progressive spermatozoa, semen volume, and testicular volume were adjusted for abstinence time; sperm concentration was also adjusted for spillage during semen sample collection; progressive spermatozoa was also adjusted for time from ejaculation to semen analysis; and reproductive hormones were also adjusted for time of day of blood sampling. cAdjusted β-coefficient for PFOS modeled as a continuous variable in a multivariable linear regression model of ln-transformed outcomes, with adjustment for covariates as indicated above and p-value as a test of linear trend.										

## Discussion

To the best of our knowledge, this is the first longitudinal study to report associations between *in utero* exposure to PFOA and semen quality and reproductive hormone levels in adult men. We observed trends of lower sperm concentration and total sperm count and higher FSH and LH with higher *in utero* exposure to PFOA. Estimates did not support associations between *in utero* exposure to PFOS and any of the investigated parameters.

For sperm concentration, total sperm count, and LH, there were no statistically significant associations with PFOA in nonparametric models based on the crude, untransformed outcome data; however, we observed significant associations after transforming the outcome data to obtain a normal distribution of residuals and adjusting for potential confounders. Adjustment of the transformed data for potential confounders did not alter the strength of the associations [see Supplemental Material, Table S4 (http://dx.doi.org/10.1289/ehp.1205118)], indicating that it was the transformation required to perform parametric linear regression analysis rather than adjustment that caused findings to differ between crude and transformed and adjusted data.

A major strength of our study is the longitudinal design, which enabled us to estimate effects of PFOA and PFOS exposure during the crucial prenatal period of male reproductive-organ programming and development on markers of adult reproductive capacity. Because correlations between maternal serum levels of PFOA and PFOS during pregnancy and offspring cord blood levels are generally good (*r* > 0.82 for PFOA and *r* > 0.72 for PFOS) (Fei et al. 2007; Gützkow et al. 2012) and because PFAA measurements from different trimesters have been shown to be highly correlated (*r* = 0.88 for PFOA and *r* = 0.87 for PFOS) (Fei et al. 2007), we believe that the measured exposure in pregnancy week 30 is a good proxy for *in utero* exposure.

Selection bias, which is caused by differences in characteristics between participants and nonparticipants of a study, is a potential concern because of the low participation rate (37.6%) and because former studies have shown that men with fertility problems are more likely to participate in reproduction studies (Bonde et al. 1996). However, in the present study, participants had no knowledge about their mothers PFAA exposure levels during pregnancy; median (interquartile range) maternal levels of PFOA were almost identical for sons who did not participate in follow-up [3.7 ng/mL (1.8)], for those who completed questionnaires [3.8 ng/mL (2.1)], and for those who underwent physical examinations [3.8 ng/mL (1.9)]. In addition, because of the participants’ young age and lack of reproductive experience, it is unlikely that the majority of participants had any knowledge about whether they had fertility problems or not; hence, it is unlikely that participation was related to fecundity.

Two human cross-sectional studies reported that exposure to high PFOA and PFOS combined or PFOS was negatively associated with the percentage of morphologically normal spermatozoa (Joensen et al. 2009; Toft et al. 2012), whereas in our study population, we observed no associations between *in utero* exposure to PFOA or PFOS and the percentage of morphologically normal spermatozoa. One possible explanation of these differences is that relevant time windows of exposure for effects on morphology and sperm production may differ, such that the former is influenced more by current exposures but the latter may be primarily influenced by developmental exposures. This is plausible because the capacity for sperm production later in life is determined during sexual organ development in fetal life, whereas the morphology and motility of spermatozoa are determined during sperm production in adolescence and adulthood. In addition, the association with sperm concentration and total sperm count suggests an effect of PFOA on Sertoli cell development; proliferation of Sertoli cells *in utero* is a determinant for spermatozoa development later in life (Sharpe et al. 2003), but the exact mechanism remains unclear. Furthermore, we observed a positive trend between *in utero* PFOA exposure and LH and FSH levels in adult men (Appasamy et al. 2007; de Kretser 1979; de Kretser et al. 1989; Gordetsky et al. 2012; Tuttelmann et al. 2009); this result supports the general notion that high gonadotrophin concentrations are expected to be associated with low sperm concentration and total sperm count, and corresponds with our finding that men in the high PFOA tertile had a tendency toward lower sperm concentration and total sperm count compared with men in the low tertile.

We observed negative associations between the proportion of motile spermatozoa and PFOA exposure, particularly when the outcome was measured using CASA. Semen concentration and motility results estimated using CASA differed substantially from the results of conventional semen analysis described previously (Vested et al. 2011), which may explain the apparent differences in the multivariable regression results between conventional semen analysis and CASA.

Studies on rats exposed *in utero* to ammonium PFOA have not suggested adverse effects on the male reproductive system (Butenhoff et al. 2004; York et al. 2010), and to date, few human studies have investigated possible effects of *in utero* exposure to xenobiotic compounds on male reproduction. In a study investigating early-life exposure to low doses of dioxin, Mocarelli et al. (2011) reported that associations with reproductive outcomes in adult males appeared to be related to postnatal exposure via breastfeeding rather than exposure *in utero.* Like dioxins, PFAAs are transferred from mother to child by breastfeeding, which would result in postnatal PFAA exposure that would be correlated with prenatal exposure (Haug et al. 2011). Therefore, we cannot exclude the possibility that the observed associations may be at least partially explained by effects of postnatal exposure rather than prenatal exposure.

Between 2000 and 2010, background levels of PFOA and PFOS have been declining in the Western world, where the percentage decline in geometric mean concentrations from 2000–2001 to 2010 was reported to be 76% for PFOS and 48% for PFOA (Olsen et al. 2012). However, the levels measured in 1988–1989 in the mothers of the present study participants (median of 3.8 ng/mL and 21.2 ng/mL for PFOA and PFOS, respectively) were lower than median levels in samples collected during 2003–2004 in Denmark (4.9 ng/mL and 24.5 ng/mL, respectively) (Joensen et al. 2009) and the United States (9.2 ng/mL and 32.3 ng/mL, respectively) (Raymer et al. 2012). Hence, *in utero* exposures in our study population were similar to or slightly lower than levels experienced by children born 15 years later.

Our findings suggest that the fetal male reproductive system may be sensitive to background exposure levels of PFOA. Corroboration by other studies would further support the hypothesis that PFOA may be a reproductive toxicant that may be contributing to reduced semen quality in adult men.

## Correction

In the manuscript originally published online, some of the p-values in Table 1 were incorrect. They have been corrected here.

## Conclusions

Although crude analyses only suggested a trend of higher FSH with increasing PFOA exposure, multivariate analyses suggested a trend of lower sperm concentration and total sperm count and a trend of higher LH and FSH with higher *in utero* exposure to PFOA. Prenatal exposure to PFOS was not related to any of the semen parameters, testicular volume, or reproductive hormones.

## Supplemental Material

(311 KB) PDF

## References

[r1] Appasamy M, Muttukrishna S, Pizzey AR, Ozturk O, Groome NP, Serhal P (2007). Relationship between male reproductive hormones, sperm DNA damage and markers of oxidative stress in infertility.. Reprod Biomed Online.

[r2] Bonde JP, Giwercman A, Ernst E (1996). Identifying environmental risk to male reproductive function by occupational sperm studies: logistics and design options.. Occup Environ Med.

[r3] Butenhoff JL, Kennedy GL, Frame SR, O’Connor JC, York RG (2004). The reproductive toxicology of ammonium perfluorooctanoate (APFO) in the rat.. Toxicology.

[r4] de Kretser DM (1979). Endocrinology of male infertility.. Br Med Bull.

[r5] de Kretser DM, McLachlan RI, Robertson DM, Burger HG (1989). Serum inhibin levels in normal men and men with testicular disorders.. J Endocrinol.

[r6] Emmett EA, Shofer FS, Zhang H, Freeman D, Desai C, Shaw LM (2006). Community exposure to perfluorooctanoate: relationships between serum concentrations and exposure sources.. J Occup Environ Med.

[r7] Fei C, McLaughlin JK, Tarone RE, Olsen J (2007). Perfluorinated chemicals and fetal growth: a study within the Danish National Birth Cohort.. Environ Health Perspect.

[r8] Giesy JP, Kannan K (2001). Global distribution of perfluorooctane sulfonate in wildlife.. Environ Sci Technol.

[r9] Gordetsky J, van Wijngaarden E, O’Brien J. (2012). Redefining abnormal follicle-stimulating hormone in the male infertility population.. BJU Int.

[r10] Grandjean P, Andersen EW, Budtz-Jørgensen E, Nielsen F, Mølbak K, Weihe P (2012). Serum vaccine antibody concentrations in children exposed to perfluorinated compounds.. JAMA.

[r11] Gützkow KB, Haug LS, Thomsen C, Sabaredzovic A, Becher G, Brunborg G (2012). Placental transfer of perfluorinated compounds is selective—a Norwegian Mother and Child sub-Cohort study.. Int J Hyg Environ Health.

[r12] Haug LS, Huber S, Becher G, Thomsen C (2011). Characterisation of human exposure pathways to perfluorinated compounds—comparing exposure estimates with biomarkers of exposure.. Environ Int.

[r13] Haug LS, Thomsen C, Becher G (2009). A sensitive method for determination of a broad range of perfluorinated compounds in serum suitable for large-scale human biomonitoring.. J Chromatogr A.

[r14] Inoue K, Okada F, Ito R, Kato S, Sasaki S, Nakajima S (2004). Perfluorooctane sulfonate (PFOS) and related perfluorinated compounds in human maternal and cord blood samples: assessment of PFOS exposure in a susceptible population during pregnancy.. Environ Health Perspect.

[r15] Jensen MS, Rebordosa C, Thulstrup AM, Toft G, Sørensen HT, Bonde JP (2010). Maternal use of acetaminophen, ibuprofen, and acetylsalicylic acid during pregnancy and risk of cryptorchidism.. Epidemiology.

[r16] Joensen UN, Bossi R, Leffers H, Jensen AA, Skakkebaek NE, Jørgensen N (2009). Do perfluoroalkyl compounds impair human semen quality?. Environ Health Perspect.

[r17] Kannan K, Corsolini S, Falandysz J, Fillmann G, Kumar KS, Loganathan BG (2004). Perfluorooctanesulfonate and related fluorochemicals in human blood from several countries.. Environ Sci Technol.

[r18] Kissa E (2001). Fluorinated Surfactants and Repellents. 2nd ed.. New York:Marcel Dekker.

[r19] Lau C, Anitole K, Hodes C, Lai D, Pfahles-Hutchens A, Seed J. (2007). Perfluoroalkyl acids: a review of monitoring and toxicological findings.. Toxicol Sci.

[r20] Menkveld R, Stander FS, Kotze TJ, Kruger TF, van Zyl JA (1990). The evaluation of morphological characteristics of human spermatozoa according to stricter criteria.. Hum Reprod.

[r21] Mocarelli P, Gerthoux PM, Needham LL, Patterson DG, Limonta G, Falbo R (2011). Perinatal exposure to low doses of dioxin can permanently impair human semen quality.. Environ Health Perspect.

[r22] Olsen GW, Burris JM, Ehresman DJ, Froehlich JW, Seacat AM, Butenhoff JL (2007). Half-life of serum elimination of perfluorooctanesulfonate, perfluorohexanesulfonate, and perfluorooctanoate in retired fluorochemical production workers.. Environ Health Perspect.

[r23] Olsen GW, Lange CC, Ellefson ME, Mair DC, Church TR, Goldberg CL (2012). Temporal trends of perfluoroalkyl concentrations in American Red Cross adult blood donors, 2000–2010.. Environ Sci Technol.

[r24] Olsen SF, Hansen HS, Sandström B, Jensen B (1995a). Erythrocyte levels compared with reported dietary intake of marine n-3 fatty acids in pregnant women.. Br J Nutr.

[r25] Olsen SF, Hansen HS, Secher NJ, Jensen B, Sandström B (1995b). Gestation length and birth weight in relation to intake of marine n-3 fatty acids.. Br J Nutr.

[r26] Palmer JR Herbst AL Noller KL Boggs DA Troisi R Titus-Ernstoff L 2009 Urogenital abnormalities in men exposed to diethylstilbestrol *in utero*: a cohort study. Environ Health 8 37; doi: [Online 18 August 2009] 10.1186/1476-069X-8-37 19689815 PMC2739506

[r27] Ramlau-Hansen CH, Thulstrup AM, Bonde JP, Ernst E (2007). Is self-measuring of testicular volume by a Prader orchidometer a valid method?. Fertil Steril.

[r28] Raymer JH, Michael LC, Studabaker WB, Olsen GW, Sloan CS, Wilcosky T (2012). Concentrations of perfluorooctane sulfonate (PFOS) and perfluorooctanoate (PFOA) and their associations with human semen quality measurements.. Reprod Toxicol.

[r29] Sharpe RM, McKinnell C, Kivlin C, Fisher JS (2003). Proliferation and functional maturation of Sertoli cells, and their relevance to disorders of testis function in adulthood.. Reproduction.

[r30] Shoeib M, Harner T, Webster GM, Lee SC (2011). Indoor sources of poly- and perfluorinated compounds (PFCS) in Vancouver, Canada: implications for human exposure.. Environ Sci Technol.

[r31] Tittlemier SA, Pepper K, Edwards L (2006). Concentrations of perfluorooctanesulfonamides in Canadian Total Diet Study composite food samples collected between 1992 and 2004.. J Agric Food Chem.

[r32] Tittlemier SA, Pepper K, Seymour C, Moisey J, Bronson R, Cao XL (2007). Dietary exposure of Canadians to perfluorinated carboxylates and perfluorooctane sulfonate via consumption of meat, fish, fast foods, and food items prepared in their packaging.. J Agric Food Chem.

[r33] Toft G, Jönsson BA, Lindh CH, Giwercman A, Spano M, Heederik D (2012). Exposure to perfluorinated compounds and human semen quality in Arctic and European populations.. Hum Reprod.

[r34] Tüttelmann F, Dykstra N, Themmen AP, Visser JA, Nieschlag E, Simoni M (2009). Anti-Müllerian hormone in men with normal and reduced sperm concentration and men with maldescended testes.. Fertil Steril.

[r35] Vested A, Ramlau-Hansen CH, Bonde JP, Thulstrup AM, Kristensen SL, Toft G (2011). A comparison of conventional and computer-assisted semen analysis (CRISMAS software) using samples from 166 young Danish men.. Asian J Androl.

[r36] Wan HT, Zhao YG, Wong MH, Lee KF, Yeung WS, Giesy JP (2011). Testicular signaling is the potential target of perfluorooctanesulfonate-mediated subfertility in male mice.. Biol Reprod.

[r37] Welsh M, Saunders PT, Fisken M, Scott HM, Hutchison GR, Smith LB (2008). Identification in rats of a programming window for reproductive tract masculinization, disruption of which leads to hypospadias and cryptorchidism.. J Clin Invest.

[r38] World Health Organization (1999). WHO Laboratory Manual for the Examination of Human Semen and Sperm–Cervical Mucus Interaction. 4th ed.

[r39] York RG, Kennedy GL, Olsen GW, Butenhoff JL (2010). Male reproductive system parameters in a two-generation reproduction study of ammonium perfluorooctanoate in rats and human relevance.. Toxicology.

