# Supplementary material for: Associations of in Utero Exposure to Perfluorinated Alkyl Acids with Human Semen Quality and Reproductive Hormones in Adult Men
Source: Environ Health Perspect. 2013 Jan 28;121(4):453–8. doi: 10.1289/ehp.1205118 (PMC3620740; doi:10.1289/ehp.1205118)
Supplement: (311 KB) PDF [file ehp.1205118.s001.pdf]

Supplemental Material

Associations of *in Utero* Exposure to Perfluorinated Alkyl Acids with Human Semen Quality and Reproductive Hormones in Adult Men

Anne Vested[1], Cecilia Høst Ramlau-Hansen[1,2], Sjurdur Frodi Olsen[3], Jens Peter Bonde[4], Susanne Lund Kristensen[1], Thorhallur Ingi Halldorsson[3, 5], Georg Becher[6], Line Småstuen Haug[6], Emil Hagen Ernst[7], Gunnar Toft[1]

[1]Danish Ramazzini Center, Department of Occupational Medicine, Aarhus University Hospital, Aarhus, Denmark

[2]Department of Public Health, Section of Epidemiology, University of Aarhus, Aarhus, Denmark

[3]Centre for Fetal Programming, Statens Serum Institut, Copenhagen, Denmark

[4]Department of Occupational and Environmental Medicine, Bispebjerg Hospital of Copenhagen University, Copenhagen, Denmark

[5]Faculty of Food Science and Nutrition, University of Iceland, Reykjavik, Iceland

[6]Division of Environmental Medicine, Norwegian Institute of Public Health, Oslo, Norway

[7]Reproductive Laboratory, Institute of Anatomy, University of Aarhus, Aarhus, Denmark

Table of Contents

Supplemental Material, Table S1 2
Supplemental Material, Table S2 3
Supplemental Material, Table S3 4
Supplemental Material Table S4 5

Supplemental Material, Table S1

Sperm concentration, total sperm count, LH, and FSH characteristics for 169 young Danish men stratified by quintiles of maternal pregnancy week 30 serum PFOA concentrations.

**Parameter**	**1st quintile:** Median (p25–p75)	**2nd quintile:** Median (p25–p75)	**3rd quintile:** Median (p25–p75)	**4th quintile:** Median (p25–p75)	**5th quintile:** Median (p25–p75)	**2nd quintile:** % difference from 1st (95% CI) [a]	**3rd quintile:** % difference from 1st (95% CI) [a]	**4th quintile:** % difference from 1st (95% CI) [a]	**5th quintile:** % difference from 1st (95% CI) [a]
Sperm conc. (mill./mL)	38 (20–62)	32 (23–51)	47 (21–95)	45 (10–70)	23 (10–47)	-2 (-46, 78)	0 (-45, 81)	-17 (-54, 52)	-43 (-69, 3)
Total sperm count (mill.)	123 (62–260)	103 (55–151)	137 (50–211)	156 (59–273)	66 (23–110)	-14 (-59, 80)	-20 (-61, 66)	-2 (-50, 93)	-55 (-77, -10)
LH	4.3 (3.6–6.1)	3.7 (3.1–4.5)	4.3 (3.0–5.1)	5.0 (4.0–6.0)	4.7 (3.6–5.6)	-4 (-24, 21)	0 (-20, 26)	24 (-1, 55)	22 (-3, 53)
FSH	2.6 (1.9–4.1)	2.7 (1.8–3.2)	3.2 (2.5–4.5)	3.4 (2.4–4.1)	3.2 (2.3–5.0)	0 (-26, 34)	22 (-9, 63)	20 (-10, 60)	39 (3, 86)

[a]Adjustment: All multivariable regression results were adjusted for history of reproductive tract disease, the son's body mass index, son's smoking status, maternal smoking during pregnancy, and socioeconomic status. Sperm concentration, and total sperm count were adjusted for abstinence time; sperm concentration was also adjusted for spillage during semen sample collection; reproductive hormones were also adjusted for time of day of blood sampling.

Note: p=percentile. Number of participants in each regression analysis depended on the outcome variable and missing data in the covariates

Supplemental Material, Table S2

CRISMAS computer assisted semen analysis (CASA) characteristics for 169 young Danish men stratified by tertiles of maternal pregnancy week 30 serum PFOA concentrations.

**Parameter**	n	Low PFOA: Median (p25–p75)	Medium PFOA: Median (p25–p75)	High PFOA: Median (p25–p75)	Spearman corr. coeff.[a]	Trend p-value[a]	Medium PFOA: % difference from low (95% CI) [b]	High PFOA: % difference from low (95% CI) [b]	Adjusted β-coeff.[c] (SE)	Adjusted trend p-value[c]
CRISMAS CASA Sperm concentration (mio/mL)	156	45 (21–60)	46 (20–88)	32 (15–62)	-0.13	0.10	-13 (-41, 29)	-33 (-54, -1)	-0.09 (0.04)	0.01
CRISMAS CASA total sperm count (mio)	114	110 (78–207)	143 (65–226)	90 (39–228)	-0.16	0.09	-7 (-43, 55)	-34 (-58, 6)	-0.18 (0.05)	<0.001
CRISMAS CASA Percentage progressive spermatozoa	162	76 (59–83)	71 (53–83)	68 (51–81)	-0.13	0.10	-9 (-20, 4)	-13 (-23, -2)	-0.03 (0.01)	0.01

[a]Spearman's rank correlation coefficient and p-value for PFOA (continuous) and untransformed outcomes.

[b]Adjustment: All multivariable regression results were adjusted for history of reproductive tract disease, the son's body mass index, son's smoking status, maternal smoking during pregnancy, abstinence time, and socioeconomic status. Sperm concentration was also adjusted for spillage during semen sample collection; progressive spermatozoa was also adjusted for time from ejaculation to semen analysis.

[c] Beta coefficient for PFOA modelled as a continuous variable in a multivariable linear regression model of ln-transformed outcomes with adjustment for covariates as indicated above, and p-value as a test of linear trend.

Note: p=percentile. Number of participants in each regression analysis depended on the outcome variable and missing data in the covariates

Supplemental Material, Table S3

CRISMAS computer assisted semen analysis (CASA) characteristics for 169 young Danish men stratified by tertiles of maternal pregnancy week 30 serum PFOS concentrations.

**Parameter**	n	Low PFOS: Median (p25–p75)	Medium PFOS: Median (p25–p75)	High PFOS: Median (p25–p75)	Spearman corr. coeff[a].	Trend p-value[a]	Medium PFOS: % difference from low (95% CI) [b]	High PFOS: % difference from low (95% CI) [b]	Adjusted β-coeff.[c] (SE.)	Adjusted trend p-value[c]
CRISMAS CASA Sperm concentration (mio/mL)	156	44 (19–65)	35 (18–65)	44 (22–97)	-0.04	0.63	-32 (-54, 0)	-7 (-38, 38)	-0.01 (0.01)	0.24
CRISMAS CASA total sperm count (mio)	114	102 (65–207)	98 (33–209)	143 (60–273)	-0.02	0.84	-46 (-66, -12)	-33 (-59, 11)	-0.02 (0.01)	0.05
CRISMAS CASA Percentage progressive spermatozoa	162	71 (56–81)	72 (50–82)	69 (55–86)	-0.05	0.50	-13 (-24, -1)	-7 (-19, 5)	-0.01 (0.00)	0.13

[a]Spearman's rank correlation coefficient and p-value for PFOS (continuous) and untransformed outcomes.

[b]Adjustment: All multivariable regression results were adjusted for history of reproductive tract disease, the son's body mass index, son's smoking status, maternal smoking during pregnancy, abstinence time, and socioeconomic status. Sperm concentration was also adjusted for spillage during semen sample collection; progressive spermatozoa was also adjusted for time from ejaculation to semen analysis.

[c] Beta coefficient for PFOS modelled as a continuous variable in a multivariable linear regression model of ln-transformed outcomes with adjustment for covariates as indicated above, and p-value as a test of linear trend.

Note: p=percentile. Number of participants in each regression analysis depended on the outcome variable and missing data in the covariates

Supplemental Material Table S4

Ln-transformed linear regression analysis results for PFOA and sperm concentration, total sperm count, LH and FSH from unadjusted (shaded) and adjusted models.

Parameter	n	Medium: % difference from low (95% CI) [b]	High: % difference from low (95% CI) [b]	β-coeff. [a] (SE)	Trend p-value [a]	Medium: Adj. % difference from low (95% CI) [b]	High: Adj. % difference from low (95% CI) [b]	Adjusted β-coeff.[c] (SE)	Adjusted trend p-value[c]
Sperm conc.	168	-9 (-42, 42)	-32 (-56, 7)	-0.10 (0.04)	0.03	-7 (-42, 47)	-34 (-58, 5)	-0.11 (0.04)	0.01
Total sperm count	123	-2 (-44; 73)	-33 (-61; 16)	-0.18 (0.06)	0.003	2 (-42, 81)	-34 (-62, 12)	-0.20 (0.06)	0.001
LH	169	7 (-10, 27)	21 (2, 44)	0.03 (0.02)	0.04	6 (-11, 27)	24 (4, 48)	0.04 (0.02)	0.03
FSH	169	18 (-5, 47)	29 (4, 61)	0.05 (0.02)	0.03	15 (-8, 44)	31(5, 64)	0.06 (0.02)	0.01

[a] Beta coefficient for PFOA modelled as a continuous variable in a linear regression model of ln-transformed outcomes, and p-value as a test of linear trend.

[b]Adjustment: All multivariable regression results were adjusted for history of reproductive tract disease, the son's body mass index, son's smoking status, maternal smoking during pregnancy, and socioeconomic status. Sperm concentration, and total sperm count were adjusted for abstinence time; sperm concentration was also adjusted for spillage during semen sample collection; reproductive hormones were also adjusted for time of day of blood sampling.

[c] Beta coefficient for PFOA modelled as a continuous variable in a multivariable linear regression model of ln-transformed outcomes with adjustment for covariates as indicated above, and p-value as a test of linear trend.
